# Effect of Levosimendan on Acute Decompensated Right Heart Failure in Patients With Connective Tissue Disease-Associated Pulmonary Arterial Hypertension

**DOI:** 10.3389/fmed.2022.778620

**Published:** 2022-03-04

**Authors:** Chao Qu, Wei Feng, Qi Zhao, Qi Liu, Xing Luo, Gang Wang, Meng Sun, Zhibo Yao, Yufei Sun, Shenglong Hou, Chunyang Zhao, Ruoxi Zhang, Xiufen Qu

**Affiliations:** ^1^Department of Cardiology, Heilongjiang Provincial People's Hospital, Harbin, China; ^2^Department of Cardiology, 1st Affiliated Hospital of Harbin Medical University, Harbin, China; ^3^Department of Cardiology, 2nd Affiliated Hospital of Harbin Medical University, Harbin, China; ^4^The Key Laboratory of Myocardial Ischemia, Ministry of Education, Harbin Medical University, Harbin, China; ^5^Department of Cardiology, Harbin 242 Hospital, Harbin, China; ^6^Department of Cardiology, Harbin Yinghua Hospital, Harbin, China

**Keywords:** levosimendan, decompensation, right heart failure, pulmonary arterial hypertension, connective tissue disease

## Abstract

**Aims:**

Acute decompensated right heart failure (RHF) in chronic precapillary pulmonary hypertension is often typified by a swiftly progressive syndrome involving systemic congestion. This results from the impairment of the right ventricular filling and/or a reduction in the flow output of the right ventricle, which has been linked to a dismal prognosis of short duration. Despite this, there are limited therapeutic data regarding these acute incidents. This study examined the effect of levosimendan on acute decompensated RHF in patients with connective tissue disease-associated pulmonary arterial hypertension (CTD-PAH).

**Methods:**

This retrospective study included 87 patients with confirmed CTD-PAH complicated acute decompensated RHF between November 2015 and April 2021. We collected biological, clinical, and demographic data, as well as therapy data, from patients with acute decompensated RHF who required levosimendan treatment in the cardiac care unit (CCU) for CTD-PAH. The patients were divided into two groups according to the levosimendan treatment. Patient information between the two groups was systematically compared in hospital and at follow-up.

**Results:**

Oxygen saturation of mixed venose blood (SvO_2_), estimated glomerular filtration rate (eGFR), 24-h urine output, and tricuspid annular plane systolic excursion (TAPSE) were found to be considerably elevated in the levosimendan cohort compared with the control cohort. Patients in the levosimendan cohort exhibited considerably reduced levels of C-reactive protein (CRP), white blood cell (WBC), troponin I, creatinine, NT-proBNP, and RV diameter compared with those in the control cohort. A higher survival rate was observed in the levosimendan cohort.

**Conclusions:**

Levosimendan treatment could effectively improve acute decompensated RHF and systemic hemodynamics in CTD-PAH patients, with positive effects on survival in hospital and can, therefore, be considered as an alternative treatment option for improving clinical short-term outcomes.

## Introduction

Pulmonary arterial hypertension (PAH) has been extensively identified as a disease condition that affects small pulmonary arteries by causing progressive vascular narrowing, resulting in increased pulmonary arterial resistance and eventual right ventricular failure (1). During the course of their condition, patients with PAH may develop acute right heart failure (RHF) (2). Acute decompensated RHF is distinguished by a rapid exacerbation of the pathological signs of RHF followed by congestion and systemic circulatory inefficiency, which could result in multisystem organ failure (3, 4). The short-term prognosis of acute decompensated RHF is extremely poor, and it continues to remain the leading cause of death among PAH patients (5, 6). Treatment of triggering factors, careful fluid management, and measures to restore cardiac function and minimize right ventricular afterload are the foundations of intensive care for acute decompensated PAH (7).

Therapeutic interventions for patients with connective tissue disease-associated pulmonary arterial hypertension (CTD-PAH) are usually more complicated than those for idiopathic PAH. In individuals with PAH linked to systemic lupus erythematosus or mixed CTD in the chronic phase, immunosuppressive treatment that combines cyclophosphamide and glucocorticoids might yield positive clinical outcomes (8), which could result in a therapeutic process similar to idiopathic PAH (9). Thus, the benefit from traditional medical therapies is somewhat lacking, stressing the importance of alternative therapeutic strategies in patients who are unable to undergo timely urgent lung transplantation and right ventricle assistance devices.

Levosimendan, a myocardial calcium sensitizer and vasodilator, which is usually utilized in left heart failure, might be effective in acute pulmonary embolism-induced right ventricular failure treatment, owing to its pharmacological profile with combined pulmonary vasodilation and increased right ventricular contractility (10). Nevertheless, there are currently insufficient data for levosimendan therapy in PAH and even acute decompensated RHF, and it is incorrect to generalize findings from trials utilizing levosimendan on the left ventricle (LV) to the RV. The focus of this study was to assess the effect of levosimendan on acute decompensated RHF in patients with CTD-PAH.

## Patients and Methods

### Study Population

Patients (>18 years) with acute decompensated RHF and a confirmed diagnosis of CTD-PAH according to the 2015 ESC/ERS Guidelines (9) were assessed for inclusion in the study. Acute RHF is described as new-onset or quickly worsening heart failure, which necessitates immediate inpatient treatment (11). The World Health Organization functional class (WHO FC) defines decompensated RHF as a condition typified by the following: (1) IV symptoms that are associated with signs of decreased blood supply to other organs (e.g., transaminase levels, lactate, troponin, and increases in creatinine); (2) clinical signs associated with venous congestion (such as liver capsule pain and neck vein congestion) with; (3) fluid retention (weight gain, ascites, and peripheral edema), resulting from decreased RV function (12). To be considered an acute decompensated RHF episode, indications of low cardiac output syndrome or volume overload necessitating vasopressor or intravenous diuretic treatment must be present. The timeframe for research started in November 2015 and lasted until April 2021. Only patients who were initially admitted to the cardiac care unit (CCU) or intensive care unit (ICU) were investigated, while recurrent hospitalizations were excluded.

### Data Collection

CCU or ICU admission was necessary for all the patients included in the study. Basic monitoring included vital signs (heart rate, blood pressure, body temperature, and O_2_ saturation), urine production, central venous pressure, central venous O_2_ saturation, and blood lactate levels. We also assessed clinical information (such as indicators of triggering factors or infections, dose regimen, treatments, heart rate, diuresis, and systemic arterial pressure), New York Heart Association functional class, echocardiographic data, and laboratory data. The laboratory data consisted of 24-h urine output, white blood cell (WBC) count, serum creatinine, oxygen saturation of mixed venose blood (SvO_2_), serum sodium, C-reactive protein (CRP), N-terminal B-type natriuretic peptide (NT-proBNP), and troponin I from the initial day of hospitalization to discharge and follow-up. The estimation of glomerular filtration rate (eGFR) on admission to the hospital was estimated utilizing the simplified Modification of Diet in Renal Disease (MDRD) algorithm: eGFR = 186.3 (SCr)^−1.154^ (age)^−0.203^ (female: ×0.742) (13).

### Echocardiography

Echocardiography was performed on patients who presented with symptoms of CTD-PAH (9). All echocardiograms were performed by expert sonographers in each location with a 2.5-MHz transducer using Vivid 5 ultrasonography equipment (GE Healthcare, Horten, Norway). All examinations underwent offline analysis by another experienced investigator at their own center. Offline assessment was conducted using commercially accessible software (EchoPac, version 8; GE Healthcare). An M-mode cursor was utilized to measure tricuspid annular plane systolic excursion (TAPSE) in the lateral tricuspid annulus from the apical 4-chamber view. Comprehensive instructions explaining the echocardiographic examination of the right heart were provided by the European Association of Cardiovascular Imaging and the American Society of Echocardiography (14, 15).

### Right Heart Catheterization

**Figure 1:**
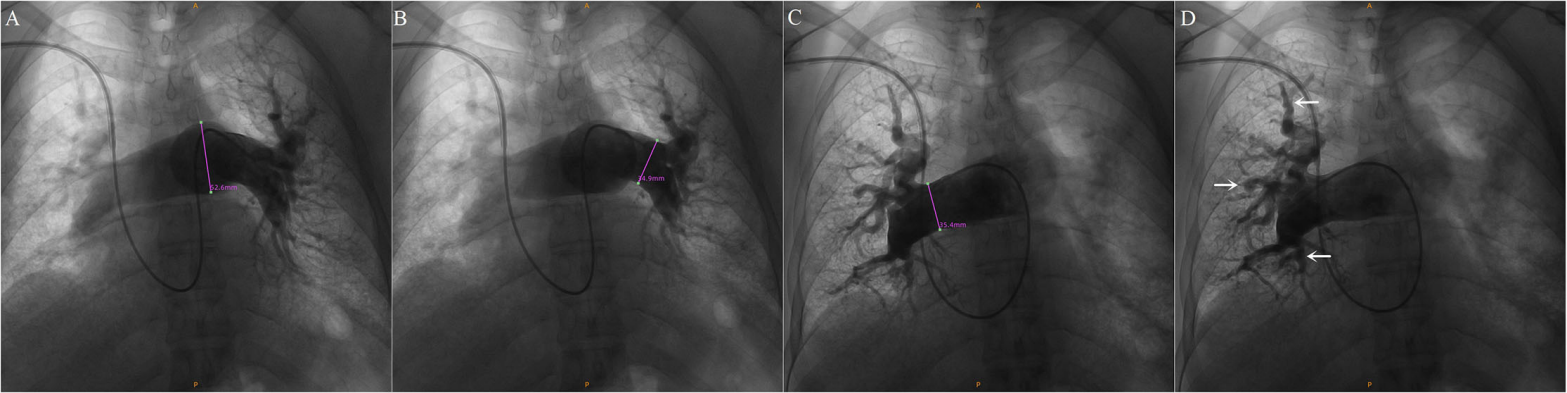
Catheter pulmonary angiogram in a 52-year-old female patient with CTD-PAH. **(A–C)** Pulmonary angiography showed that the diameter of the pulmonary artery was enlarged. **(A)** Main pulmonary artery. **(B)** Left and right pulmonary artery. **(C)** Right pulmonary artery. **(D)** Distal pulmonary artery changed into tortuousness (arrow).

Some in-hospital or hospitalized reexamination patients underwent right heart catheterization (RHC) for invasive hemodynamic and imaging measurements once their condition was stable. RHC was performed as part of the diagnostic work-up after the catheter pulmonary angiogram (Figure 1) (9). RHC was regularly performed with a 6F sheath and a conventional Swan-Ganz catheter into the right internal jugular vein. The dose or protocol of PH-targeted therapy was modified in some patients who underwent RHC assessment.

### Treatment

The main concepts of CCU/ICU care of patients with CTD-PAH and acute decompensated RHF consist of treating trigger factors (including infections, arrhythmias, anemia, or other comorbidities), optimizing fluid balance (through the use of intravenous diuretics), reducing the RV afterload, improving cardiac output (CO) with inotropes, and maintaining systemic blood pressure using vasopressors (8, 12). Due to the possibly detrimental hemodynamic effects of positive intrathoracic pressure on RV performance (16), no patient was mechanically ventilated during the research period.

The patients in the levosimendan cohort were administered levosimendan (Shengnuo Pharmaceutical Co., Ltd., Sichuan, China) 0.05 μg/kg/min which was raised to 0.1 μg/kg/min infusion for 24 h (17). The elimination half-life of levosimendan is relatively short at ~1 h, while the active metabolite OR-1896 has a 70–80-h elimination half-life, enabling hemodynamic impacts to last for 7–9 days following 24-h administration of levosimendan (18). If the patient's symptoms were not relieved after 1 week, levosimendan could be administered again. Patients in the control group received a continuous infusion of 0.5 μg/kg/min continuous infusion milrinone [Lunan Pharmaceutical (Group) Co., Ltd., Hubei, China] (19). To reduce RV afterload, Epoprostenol therapy (1 ng·kg^−1^·min^−1^) was initiated while patients were in the CCU and the dose was increased every 12 h. On day 5, a maintenance dose of 10 ng·kg^−1^·min^−1^ daily was initiated.

### Statistical Analysis

Quantitative data are expressed as the mean value ± standard deviation, while qualitative data are expressed as frequency (percentage). The independent two-sample *t*-test was used for comparisons between cohorts. The chi-square test or Fisher's exact test was used to compare categorical variables, as applicable. Cox model was used to evaluate the association between patients' characteristics and in-hospital mortality and was adjusted for all other baseline characteristics with p <0.10 on univariable analysis. To estimate survival status, the Kaplan–Meier (KM) technique was utilized, and the log-rank test was used to compare survival distributions. Two-sided *P*-values of <0.05, were deemed statistically significant. SPSS version 19.0 (SPSS Inc., Chicago, IL, USA) was used to conduct all statistical analyses.

## Results

### Baseline Characteristics

In this retrospective study, 87 patients with confirmed CTD-PAH complicated with acute decompensated RHF were enrolled. Baseline and RHC parameters did not differ significantly (Table 1).

**Table 1:** Baseline characteristics of patients hospitalized with acute decompensated right heart failure.

	**Levosimendan group** **(*n* = 46)**	**Control group** **(*n* = 41)**	***P*** **-value**
Age, years	55.52 ± 10.35	53.20 ± 8.83	0.261
Women, *n* (%)	40 (86.96)	37 (90.24)	0.743
**Primary disease**			
Systemic sclerosis	14 (30.43)	9 (21.95)	0.467
Systemic lupus erythematosus	18 (39.13)	23 (56.10)	0.135
Sjogren's syndrome	9 (19.57)	6 (14.63)	0.583
Rheumatoid arthritis	5 (10.87)	3 (7.32)	0.717
**Triggering factors of RHF**			
Infection	31 (67.39)	29 (70.73)	0.818
Arrhythmia	9 (19.57)	6 (14.63)	0.583
Emotion	1 (2.17)	2 (4.88)	0.600
Exhaustion	1 (2.17)	2 (4.88)	0.600
Discontinuing medication	4 (8.70)	2 (4.88)	0.680
**Vital signs**			
Systolic blood pressure, mmHg	102.93 ± 15.51	104.12 ± 12.78	0.697
Heart rate, beats/min	95.83 ± 10.16	93.76 ± 9.16	0.320
Respiratory rate, breaths/min	22.46 ± 3.21	22.90 ± 4.81	0.609
**PH-targeted therapy before admission**			
Beraprost	20 (43.48)	20 (48.78)	0.670
Sildenafil	17 (36.96)	15 (36.59)	1.000
Bosentan	13 (28.26)	15 (36.59)	0.492
**Laboratory testing**			
SvO_2_, %	49.72 ± 9.22	49.05 ± 9.03	0.734
WBC, ×10^9^/L	13.45 ± 4.90	14.44 ± 3.64	0.285
CRP, mg/L	22.85 ± 12.40	25.93 ± 9.96	0.203
NT-proBNP, pg/mL	5903.50 ± 2480.97	5312.93 ± 2517.21	0.275
Troponin I, μg/L	1.14 ± 0.67	1.30 ± 0.76	0.331
Creatinine, μmol/L	145.41 ± 43.42	148.71 ± 50.27	0.746
eGFR, mL/min/1.73 m^2^	40.17 ± 14.40	39.12 ± 12.53	0.717
Serum sodium, mmol/L	142.21 ± 1.95	142.23 ± 0.34	0.951
**Echocardiographic parameters**			
RV size, mm	39.07 ± 6.64	39.84 ± 7.35	0.612
RV hypokinesis, *n* (%)	26 (56.52)	24 (58.54)	1.000
TAPSE, cm	2.10 ± 0.92	2.11 ± 0.69	0.948

*Mean values (standard deviation) and % (n) were reported for continuous and categorical variables, respectively. RHF, right heart failure; SvO_2_, oxygen saturation of mixed venose blood; WBC, white blood cell; CRP, C-reactive protein; NT-proBNP, N-terminal pro-B type natriuretic peptide; eGFR, estimated glomerular filtration rate; TAPSE, tricuspid annular plane systolic excursion; RV, right ventricle*.

### Right Heart Catheterization

**Figure 2:**
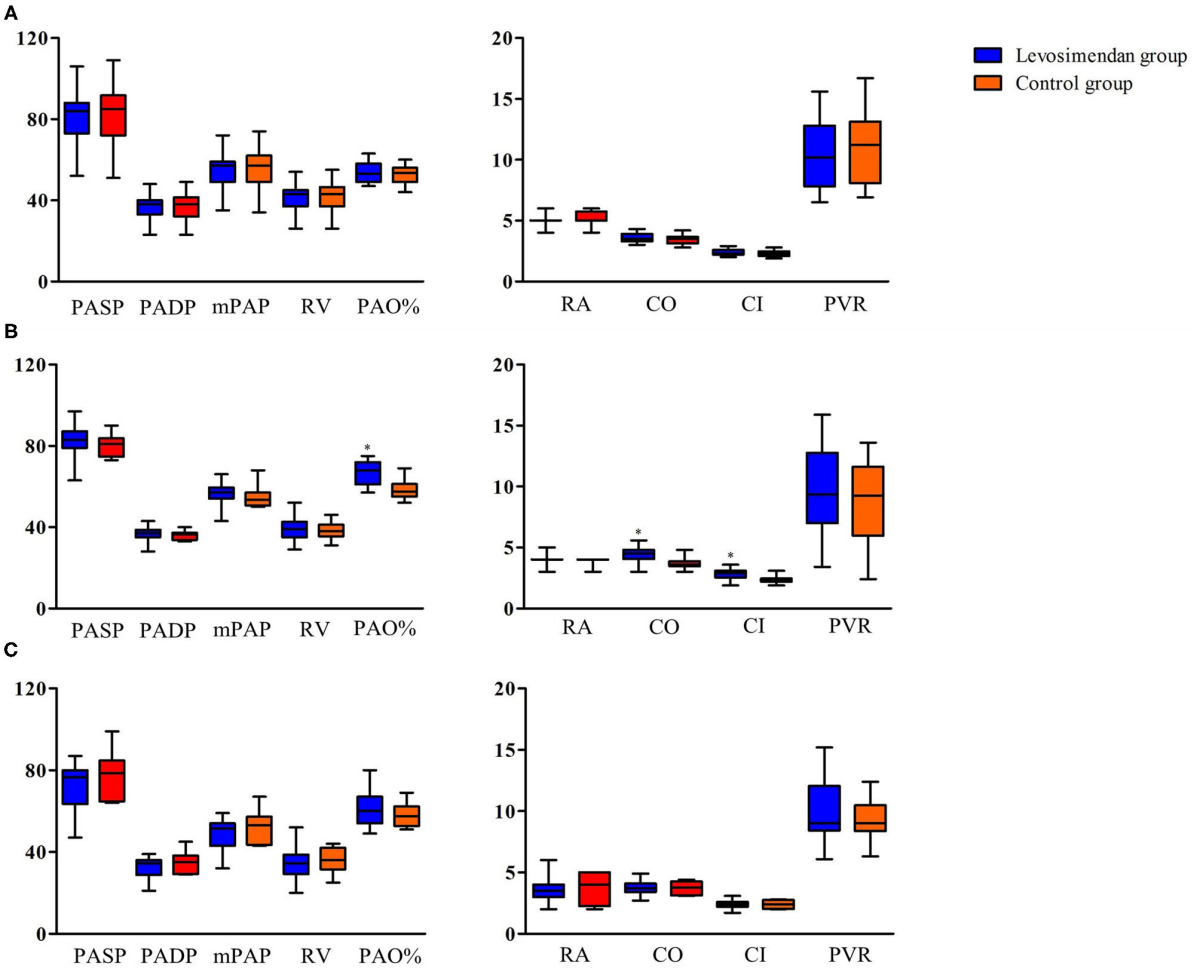
Invasive hemodynamic measurements in patients with right heart catheterization. **(A)** On admission. **(B)** After drug treatment and symptom relief. **(C)** In the first 6 months after discharge. PASP, pulmonary artery systolic pressure; PADP, pulmonary artery diastolic pressure; mPAP, mean pulmonary artery pressure; RA, right atrial mean pressure; RV, right ventricular mean pressure; PAO, pulmonary arterial oxygen saturation; CO, cardiac output; CI, cardiac index; PVR, pulmonary vascular resistance; *P < 0.05 between levosimendan and control groups.

Of the 87 patients, only 35 underwent right cardiac catheterization on admission. There were no differences in the RHC on admission (Figure 2A). Forty-two patients received right cardiac catheterization after drug treatment and symptom relief, due to hemodynamic instability caused by decompensated right heart failure at admission. Levels of PAO%, CO, and CI were significantly higher in the levosimendan group than in the control group (Figure 2B). Ten patients died before right cardiac catheterization was performed due to uncorrectable right heart failure. Right cardiac catheterization was performed in 28 patients in the first 6 months after discharge. No significant difference was observed between two groups (Figure 2C).

### Patients' Characteristics at Discharge

**Figure 3:**
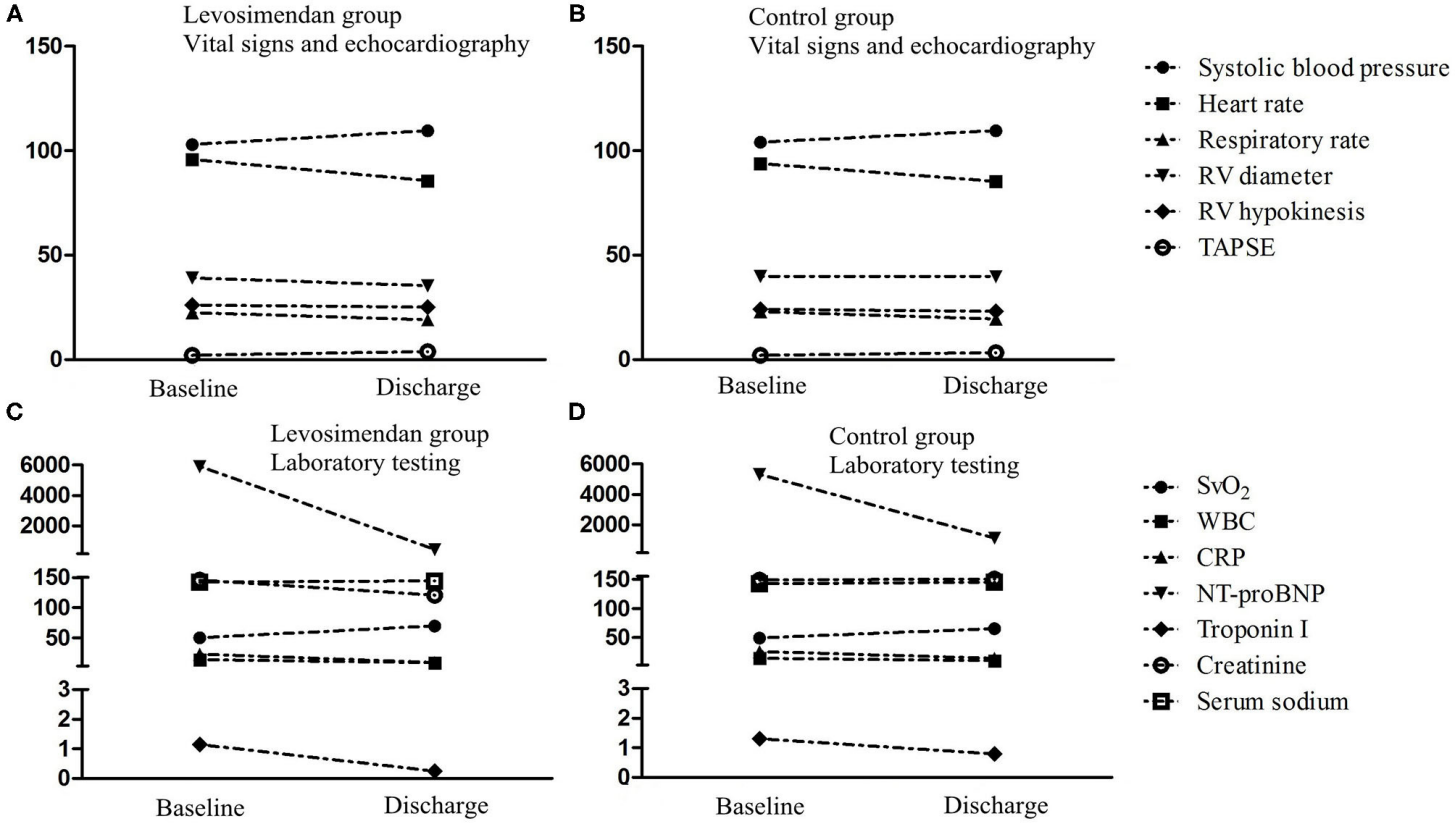
Changes of clinical characteristics from baseline to discharge. SvO_2_, oxygen saturation of mixed venose blood; WBC, white blood cell; CRP, C-reactive protein; NT-proBNP, N-terminal pro-B type natriuretic peptide; eGFR, estimated glomerular filtration rate; TAPSE, tricuspid annular plane systolic excursion; RV, right ventricle; RVSP, right ventricular systolic pressure. **(A)** Vital signs and echocardiography in levosimendan group. **(B)** Vital signs and echocardiography in control group. **(C)** Laboratory testing in levosimendan group. **(D)** Laboratory testing in control group.

The patient characteristics at discharge are shown in Table 2. SvO_2_, eGFR, 24-h urine output, and TAPSE were found to be considerably elevated in the levosimendan cohort compared with the control cohort. Additionally, the patients in the levosimendan cohort exhibited considerably decreased levels of WBC, CRP, troponin I, creatinine, NT-proBNP, and RV diameter compared with the control group. Changes in the clinical characteristics from baseline to discharge are shown in Figure 3.

**Table 2:** Comparison of patients' characteristics at discharge.

	**Levosimendan group** **(*n* = 42)**	**Control group** **(*n* = 29)**	***P*** **-value**
**Vital signs**			
Systolic blood pressure, mmHg	109.60 ± 11.24	109.59 ± 16.22	0.998
Heart rate, beats/min	85.69 ± 3.98	85.31 ± 7.69	0.789
Respiratory rate, breaths/min	19.12 ± 1.70	19.48 ± 4.72	0.647
**Laboratory testing**			
SvO_2_, %	69.26 ± 3.76	65.28 ± 6.28	0.001
WBC, ×10^9^/L	8.43 ± 2.34	10.45 ± 3.57	0.005
CRP, mg/L	8.72 ± 4.86	14.39 ± 9.08	0.001
Creatinine, μmol/L	120.48 ± 22.88	150.17 ± 36.48	<0.001
eGFR, mL/min/1.73 m^2^	46.12 ± 3.76	37.24 ± 12.35	0.003
NT-proBNP, pg/mL	430.79 ± 188.66	1130.31 ± 534.06	<0.001
Troponin I, μg/L	0.24 ± 0.15	0.79 ± 0.30	<0.001
Serum sodium, mmol/L	143.99 ± 3.83	144.90 ± 3.88	0.332
24-h urine output, ml/day	1338 ± 236	1105 ± 195	<0.001
**Echocardiographic parameters**			
RV diameter, mm	35.29 ± 5.89	39.70 ± 10.98	0.032
RV hypokinesis, *n* (%)	25 (59.52)	23 (79.31)	0.121
TAPSE, cm	3.81 ± 0.72	3.36 ± 0.71	0.010

*Mean values (standard deviation) and % (n) were reported for continuous and categorical variables, respectively. SvO_2_, oxygen saturation of mixed venose blood; WBC, white blood cell; CRP, C-reactive protein; NT-proBNP, N-terminal pro-B type natriuretic peptide; eGFR, estimated glomerular filtration rate; TAPSE, tricuspid annular plane systolic excursion; RV, right ventricle; RVSP, right ventricular systolic pressure*.

### Clinical Events During Hospitalization and in the First 6 Months After Discharge

**Figure 4:**
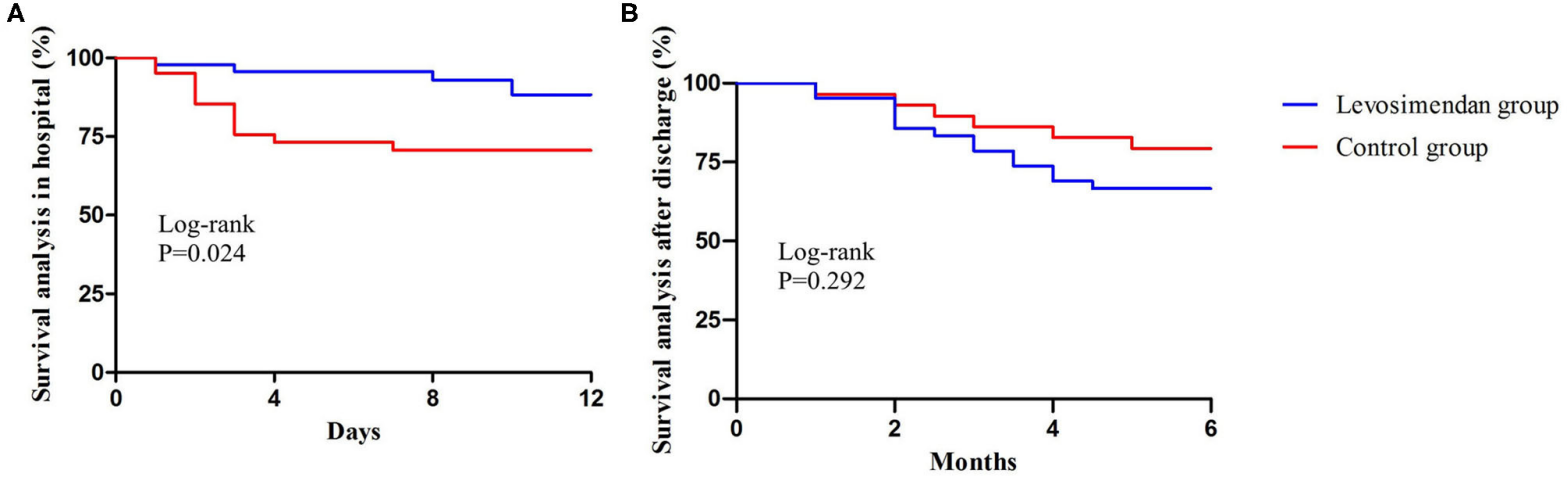
Survival analysis between two groups in hospital **(A)** and after discharge **(B)**.

In hospitals, the all-cause mortality and death from RHF were considerably lower in the levosimendan cohort than in the control cohort (8.7% vs. 29.27%, *P* = 0.024 and 4.35% vs. 21.95%, *P* = 0.021; Table 3 and Figure 4A). In the first 6 months after discharge, no significant difference was identified in the survival rate between the two cohorts (Table 3 and Figure 4B). Univariate Cox regression analysis indicated that levosimendan (hazard ratio = 0.230, *P* = 0.019), Troponin I (hazard ratio =1.320, *P* = 0.043) and SvO_2_ (OR = 0.609, *P* = 0.001) were associated with all-cause mortality in hospitals (Table 4). Levosimendan (hazard ratio, 0.186; *P* = 0.007) and SvO_2_ (hazard ratio, 0.768; *P* < 0.001) were independently associated with in-hospital mortality in the multivariate Cox regression analysis (Table 4).

**Table 3:** Clinical events during hospitalization and in the first 6 months after discharge.

	**Levosimendan group** **(*n* = 46)**	**Control group** **(*n* = 41)**	***P*** **-value**
**In-hospital**			
All-cause mortality	4 (8.70)	12 (29.27)	0.024
Death from RHF	2 (4.35)	9 (21.95)	0.021
**In the first 6 months after discharge**			
All-cause mortality	18 (39.13)	18 (43.90)	0.669
Death from RHF	11 (23.91)	14 (34.15)	0.347
Rehospitalization associated with RHF	25 (54.35)	24 (58.54)	0.829

*Mean values (standard deviation) and % (n) were reported for continuous and categorical variables, respectively. RHF, right heart failure*.

**Table 4:** Univariate and multivariate Cox regression analysis for in-hospital death.

	**Univariate**		**Multivariate**	
	**Hazard ratio (95% CI)**	***P*** **-value**	**Hazard ratio (95% CI)**	***P*** **-value**
Levosimendan	0.230 (0.068–0.785)	0.019	0.186 (0.055–0.630)	0.007
SvO_2_	0.609 (0.458–0.809)	0.001	0.768 (0.695–0.849)	<0.001
CRP	0.986 (0.943–1.031)	0.540	–	–
eGFR	0.972 (0.935–1.011)	0.161	–	–
Troponin I	1.320 (1.062–2.631)	0.043	0.762 (0.353–1.645)	0.489
NT-proBNP	1.000 (1.000–1.000)	0.076	1.000 (1.000–1.000)	0.024
TAPSE	1.007 (0.550–1.844)	0.981	–	–

*CI, confidence interval; SvO_2_, oxygen saturation of mixed venous blood; CRP, C-reactive protein; NT-proBNP, N-terminal pro-B type natriuretic peptide; eGFR, estimated glomerular filtration rate; TAPSE, tricuspid annular plane systolic excursion*.

## Discussion

Our findings indicate that in the present treatment period, acute decompensated RHF is still a severe life-threatening disease. Our data demonstrated that levosimendan could improve clinical indices and survival rates during hospitalization, but had no significant effect on medium-and long-term outcomes at follow-up.

It is widely established that people with CTD are more likely to develop group 1 PAH (9). Preclinical research supports the idea that PAH may be caused by immunological dysfunction (20). CTD-PAH is typified by vasoconstriction and modification of small-to-medium-sized pulmonary arterioles, as well as significant interstitial inflammation and fibrotic alterations (21). Patients with CTD-PAH have a worse overall survival rate than patients diagnosed with other forms of PAH. Greater awareness of PAH morbidity and death in CTDs has resulted in a growing number of research studies investigating disease burden, pathophysiology, and risk factors related to the development of PAH in CTDs. Current therapeutic guidelines and updates mainly focus on generalized treatment for all group 1 PAH without any specific guidelines for managing CTD-PAH (22, 23). The current treatment algorithm may be divided into three main areas: (1) general measures, supportive therapy, referral strategy, acute vasoreactivity testing, and chronic treatment with calcium channel blockers; (2) initial therapy with approved PAH drugs; and (3) clinical response to the initial therapy, combination therapy, balloon atrial septostomy, and lung transplantation (22). Therefore, it is worth exploring alternative therapies for acute decompensated RHF in patients with CTD-PAH (23).

Levosimendan is recognized as a calcium-sensitizing agent comprising cardioprotective, pulmonary vasodilatory, and inotropic characteristics. In contrast to other inotropes, levosimendan's positive inotropic effects do not occur at the cost of elevated myocardial oxygen demand or calcium overload (24, 25). As previous studies have shown, levosimendan has slightly distinct effects on the left and right ventricular hemodynamic parameters. In the systemic vasculature, the vasodilator response is greater than the response in the pulmonary circulation (26). On the other hand, the positive inotropic impact is larger on the right than on the left ventricles, as indirectly evaluated by the percentage increase in stroke index (18). This could imply that an increase in right contractility instead of a reduction in afterload is the main mechanism of action of levosimendan in right ventricular dysfunction. This may lead to the emergence of a particularly high-risk group of patients requiring additional vasopressor treatment. We cannot rule out that the vasodilating effects of levosimendan in these high-risk individuals may worsen the detrimental consequences of hypotension and the toxic effects of vasopressor treatment. Thus, caution should be exercised while administering levosimendan, and a loading dosage should be prevented.

It is worth noting that our study showed no significant differences in the RHC characteristics at follow-up. Some studies indicated that levosimendan could enhance RV function, enhance RV mechanical efficiency, improve clinical symptoms, and reduce pulmonary arterial pressure in treating patients with acute decompensated RHF due to PAH. However, the improved clinical effect caused by levosimendan may disappear when exceeding the time of hemodynamic persistent effects, 7–9 days as reported (18). Further research in future trials with a larger patient population and a longer follow-up period is required.

Right hemodynamic monitoring by RHC has been identified as the most efficient tool for evaluating cardiac function, right ventricular preload, and right ventricle afterload in pulmonary hypertension. The primary modified parameters in acute decompensation are right atrial pressure and cardiac output, which have been shown to be significant prognostic indicators of PAH (27). Nevertheless, invasive hemodynamic monitoring may be hazardous in severely unstable PAH patients, which poses a risk of arrhythmia and infection. Studies have shown that echocardiography may be a safe method for obtaining essential data on systolic pulmonary arterial pressure and cardiac index as an appropriate reflection of right ventricular preload (28, 29). After analyzing the benefit-to-risk ratio, RHC is recommended in severe and complicated cases (3). Meanwhile, the central venous line might be beneficial in monitoring the central venous pressure to estimate the development of right ventricular preload and improve fluid balance management with diuretics. Furthermore, central venous oxygen saturation measurements are advised to assess tissue oxygenation (3).

In our study, NT-proBNP, troponin I, and CRP levels in the levosimendan cohort were significantly reduced at discharge compared with the control group. Several studies have verified the prognostic significance of biomarkers such as troponin and BNP (5, 30). In individuals with acute decompensated pulmonary hypertension, CRP levels are helpful in screening for inflammatory processes (5), probably indicating a systemic inflammatory reaction syndrome due to acute right cardiac decompensation. Numerous studies have confirmed the inflammatory reaction in acute RHF and indicated the probable diagnostic and prognostic values of these inflammatory markers in such cases (31). It has also been shown to have prognostic significance when coupled with RV dysfunction, a predictor of poor PAH results (32). Inflammatory activation is directly related to the extent of impairment of cardiac function and neurohormonal activation. Inflammatory pathways emphasize that additional research on stable and decompensated PAH is necessary to discover new treatment targets. There have been increasing efforts to understand the relationship between autoimmunity, inflammation, and the evolution of PAH (32). In the latest research, Immunosuppressive therapy of rituximab in patients with systemic sclerosis-pulmonary arterial hypertension (SSc-PAH) was associated with inflammatory resolution following B-cell depletion (33).

Our results further indicate that patients with acute decompensated RHF should preferably be treated in specialist facilities that can access all interventional, medical, and surgical alternatives, including lung transplantation, ECMO, and medical therapies. Identifying alternative treatment strategies in patients with PAH and acute decompensated RHF is essential for improving outcomes.

### Limitations

First, the research included only a limited number of individuals, carefully selected based on illness severity and etiology. Second, since this was not a randomized controlled trial (RCT), the findings cannot be contrasted directly to a levosimendan-based therapeutic strategy. Third, during hospitalization, right ventricular performance was assessed semi-quantitatively using echocardiography; direct measurement of the right cardiac catheter could have improved the relevance of the study. Large RCTs are required in order to acquire additional long-term information regarding the prognosis and survival rate of patients with decompensated RHF receiving levosimendan treatment.

## Conclusion

Our results show that levosimendan treatment is practicable and enhances hemodynamic parameters during hospitalization in individuals with decompensated RHF. The increase in the right ventricular stroke index, as well as the leftward shift of the association between the right ventricular preload and cardiac output, illustrated that the surge in the contractility of the right ventricle instead of the reduction of the afterload is the key underlying mechanism. To validate this notion, further research involving a larger number of patients is required.

## Data Availability Statement

The datasets presented in this study can be found in online repositories. The names of the repository/repositories and accession number(s) can be found at: https://pan.baidu.com/s/1tsHlgG0RLcm8AZfhitJCHw.

## Ethics Statement

The studies involving human participants were reviewed and approved by Ethics Committee for the First Affiliated Hospital of Harbin Medical University. The patients/participants provided their written informed consent to participate in this study. Written informed consent was obtained from the individual(s) for the publication of any potentially identifiable images or data included in this article.

## Author Contributions

CQ, WF, RZ, and XQ conceived and designed the experiments. CQ, WF, QZ, XL, GW, ZY, YS, and SH performed the experiments. CQ, WF, MS, QL, CZ, and RZ analyzed the data. CQ, WF, and RZ wrote the manuscript. All authors contributed to the manuscript and approved the submitted version.

## Funding

This work was supported by the Key Laboratory of Myocardial Ischemia, Chinese Ministry of Education, Harbin, Heilongjiang Province, China [Grant Number KF201823 (to CQ)].

## Conflict of Interest

The authors declare that the research was conducted in the absence of any commercial or financial relationships that could be construed as a potential conflict of interest.

## Publisher's Note

All claims expressed in this article are solely those of the authors and do not necessarily represent those of their affiliated organizations, or those of the publisher, the editors and the reviewers. Any product that may be evaluated in this article, or claim that may be made by its manufacturer, is not guaranteed or endorsed by the publisher.
